# Combining clinical and molecular heterogeneity within CASPR2-antibody mediated diseases: towards the underlying disease biology

**DOI:** 10.1136/jnnp-2020-323457

**Published:** 2020-07-10

**Authors:** Sophie Binks, Sarosh R Irani

**Affiliations:** Oxford Autoimmune Neurology Group, Nuffield Department of Clinical Neurosciences, University of Oxford, Oxford, UK

**Keywords:** autoimmune encephalitis, neuroimmunology, clinical neurology, immunology, neurogenetics

## Muñiz-Castrillo aims towards precision medicine in CASPR2-antibody diseases by dissecting clinical features, serology and genetics across 56 patients.

Since their description in 2010,^1^ the spectrum of clinical features associated with CASPR2-antibodies has evolved. Today, cramps, neuropathic pain, hyperhidrosis, hypertension, seizures, cognitive impairment, psychiatric features and cerebellar ataxia are all recognised associations.^2–4^ This clinical diversity has intensified with the interweaving of increasingly broad observations regarding patient demographics and tumour status.^3^ One method to deconvolute clinical boundaries is to use the fundamental biology as a gold standard for classification. In the paper by Muñiz-Castrillo, the authors explain the complexity underlying the CASPR2-antibody diseases using a data-driven approach to phenotyping, in parallel with immunological and genetic markers integral to disease pathogenesis.^5^

By cluster analysis, 56 patients with CASPR2-antibodies were divided into four groups: (i) a relatively isolated limbic encephalitis (LE) (‘LE/-’; n=18), (ii) LE ‘plus’ non-limbic symptoms, including cerebellar ataxia, movement disorders, dysautonomia and weight loss (‘LE/+’; n=11), (iii) a group with severe peripheral nerve hyperexcitability (PNH), plus dysautonomia, dyssomnia and malignant thymoma, but without limbic features—closely resembling contemporary descriptions of Morvan’s syndrome (‘PNH/+’; n=16)^2^ and (iv) a less severe form of PNH with only few associated central nervous system (CNS) features (‘PNH/-’; n=11). These phenotypes explained some of the variance observed across paraclinical findings, such as inflammation on MRI/cerebrospinal fluid (CSF). Yet, it is unclear whether these groups will accommodate all CASPR2-antibody presentations, such as those who present with limited cognitive impairment but a prominent movement disorder.

Nevertheless, these groups showed marked differences in autoantibody profiles. For example, CSF CASPR2-antibodies were detected in 27/29 patients from the two LE groups but only 4/7 with PNH/- and, perhaps surprisingly, 0/12 with PNH/+. Such absence of detectable CSF CASPR2-antibodies in patients with CNS-localisable features may represent an emerging theme in the field.^3^ A similar trend was observed for serum CASPR2-antibody levels: these were highest in both LE groups>PNH/+>PNH/-. Co-existent LGI1-antibodies were only detected in the PNH/+ patients with a malignant thymoma, a finding which may usefully help accurately identify a paraneoplastic subgroup.^2 6^

Finally, the human leucocyte antigen (HLA) genotypes of 30 patients were analysed and yielded the most categorical result. HLA-DRB1*11:01 was first identified in 2018 as a risk allele in 15/31 (48%) of patients with CASPR2-antibodies, alongside an extended haplotype.^6^ Now, Muñiz-Castrillo et al show HLA-DRB1*11:01 is present in 93% (16/17) of genotyped LE cases (both LE/- and LE/+), but observed at rates equivalent to healthy controls in the PNH/+ and PNH/- patients. Hence, with a frequency akin to HLA-DRB1*07:01 in patients with LGI1-antibodies,^6^ a single allelic HLA association of CASPR2-antibodies in CNS diseases may almost be the rule.^7^ This finding now awaits confirmation: analysis of our published cases showed a less striking bias given 13/22 (59%) with exclusive CNS features carried HLA-DRB1*11:01, compared with 2/5 (40%) with PNH^+/-^.

Despite incomplete data sets for some patients, Muñiz-Castrillo et al illustrate a simple yet elegant method to use the fundamental biology to (re)define the clinical syndrome. The principal serological and genetic segregations were observed between patients with and without PNH. This ‘take home point’ largely returns to traditional CASPR2-antbody associated syndromic categorisations: PNH, Morvan’s syndrome and LE. Moreover, it suggests that the mechanism of the autoimmunisation required to generate CASPR2-antibodies may determine the clinical manifestations and the presence or absence of CSF CASPR2-antibodies. More practically, adjunctive HLA-testing could help introduce precision-medicine to the autoimmune neurology clinic and reinforce observations suggesting genotyping might indicate which CASPR2-antibody patients will be most immunotherapy-responsive.^6^

In conclusion, a fundamental immunogenetic distinction may underlie the localisation of CASPR2-antibody diseases. This important new insight is reminiscent of the reclassification prompted by refining the biochemistry which identified LGI1-antibodies and CASPR2-antibodies from the now clinically-redundant VGKC-antibody assay.^8^ Muñiz-Castrillo’s finding should usher in fruitful new understanding of the biology of CASPR2-antibody diseases, with future implications for pathogenesis and selection of immunotherapies.
